# Structural gray matter differences in Problematic Usage of the Internet: a systematic review and meta-analysis

**DOI:** 10.1038/s41380-021-01315-7

**Published:** 2021-10-12

**Authors:** Jeremy E. Solly, Roxanne W. Hook, Jon E. Grant, Samuele Cortese, Samuel R. Chamberlain

**Affiliations:** 1 Cambridge University Hospitals NHS Foundation Trust, Cambridge, UK; 2 Department of Psychiatry, University of Cambridge, Cambridge, UK; 3 Department of Psychiatry, University of Chicago, Pritzker School of Medicine, USA; 4 Centre for Innovation in Mental Health, School of Psychology, Faculty of Environmental and Life Sciences, University of Southampton, Southampton, UK; 5 Clinical and Experimental Sciences (CNS and Psychiatry), Faculty of Medicine, University of Southampton, Southampton, UK; 6 Solent NHS Trust, Southampton, UK; 7 Hassenfeld Children’s Hospital at NYU Langone, New York University Child Study Center, New York City, New York, USA; 8 Division of Psychiatry and Applied Psychology, School of Medicine, University of Nottingham, Nottingham, UK; 9 Southern Health NHS Foundation Trust, Southampton, UK; 10 Cambridgeshire and Peterborough NHS Foundation Trust, Cambridge, UK

## Abstract

Problematic Usage of the Internet (PUI) has been linked to diverse structural gray matter changes in individual data studies. However, no quantitative synthesis across studies has been conducted. We aimed to identify gray matter regions showing significant spatial convergence across neuroimaging studies in PUI. We searched PubMed and PsycINFO up to 10/03/2021 and included original, cross-sectional comparative studies that examined structural gray matter imaging in PUI versus control groups; reported a whole brain analysis; and provided peak coordinates for gray matter differences. From a total of 624 potentially relevant studies, 15 (including 355 individuals with PUI and 363 controls) were included in a meta-analysis of voxel-based morphometry studies. Anatomical likelihood estimation (ALE) meta-analysis was performed using extracted coordinates and identified significant spatial convergence in the medial/superior frontal gyri, the left anterior cingulate cortex/cingulate gyrus and the left middle frontal/precentral gyri. Datasets contributing to these findings all indicated reduced gray matter in cases compared to controls. In conclusion, voxel-based morphometric studies indicate replicable gray matter reductions in the dorsolateral prefrontal cortex and anterior cingulate cortex in PUI, regions implicated in reward processing and top-down inhibitory control. Further studies are required to understand the nature of gray matter differences across PUI behaviors, as well as the contribution of particular mental health disorders, and the influence of variation in study and sample characteristics.

## Introduction

Although the Internet plays a useful role in many areas of life, a subset of Internet users develop Problematic Usage of the Internet (PUI), characterized by loss of control and adverse consequences, such as feelings of distress and functional impairment in daily life [1]. PUI is a broad term that refers to a range of excessive online activities, including general surfing, gaming, gambling, buying/shopping, pornography use and social networking [2, 3]. It has been linked to impaired functioning, high rates of psychiatric morbidity and reduced quality of life [4–6]. Though operational definitions vary, meta-analytic evidence indicates a high prevalence of PUI and that rates appear to be escalating over time [7]. Thus, PUI is a growing public health concern.

Currently, gaming disorder and gambling disorder are included in the ICD-11 as disorders that may involve both online and offline behavior [8], while Internet Gaming Disorder (IGD) is included in the Diagnostic and Statistical Manual of Mental Disorders Version 5 (DSM-5) as a condition for further study [9]. Other examples of problematic Internet-related behaviors, such as social networking [10], are not currently included in classification systems. There is ongoing discussion regarding whether these behaviors are best classified using the concept of PUI/Internet addiction or as specific addictions to various Internet-related activities [11]. Despite the heterogeneity of PUI, a meta-analysis of cognitive deficits in PUI found that, irrespective of whether gaming was the predominant type of online behavior, PUI was characterized by pronounced impairment on cognitive domains which are known to be related to the fronto-striatal brain circuitry with small-medium effect sizes [12]. Further research is needed to understand the similarities and differences between different forms of PUI and between PUI and other mental disorders [3].

Whilst reproducible results have been reported in the neuropsychological literature, gray matter structural brain abnormalities have been variably associated with PUI, including in areas implicated in reward processing and top-down inhibitory control [13–18]. However, there are many discrepancies between published studies, which have found inconsistent abnormalities across diverse regions of the brain [13, 18]. As neuroimaging literature grows, meta-analysis becomes an important tool to synthesize findings across studies [19]. Prior meta-analyses focusing on IGD using signed differential mapping identified lower gray matter volume in the anterior cingulate cortex (ACC), supplementary motor area (SMA), right putamen, right inferior frontal gyrus and left dorsolateral prefrontal cortex (DLPFC) [20, 21]. However, meta-analysis of gray matter structural studies across the PUI literature has yet to be conducted.

Therefore, the aim of this study was to conduct the first systematic review and meta-analysis of gray matter structural imaging studies of PUI. We used anatomical likelihood estimation (ALE) methodology, which is a meta-analytic technique focusing on spatial convergence of foci rather than effect sizes [22–24]. Based on findings from neuropsychological studies in PUI, we hypothesized that PUI would be associated with abnormal gray matter in neural regions implicated in reward processing and top-down inhibitory control.

## Materials and Methods

The protocol for this study was pre-registered in the international prospective register of systematic reviews (PROSPERO 2020 CRD42020176234), which also indices any subsequent changes made to the protocol with a detailed rationale (Supplementary Methods 1). Study reporting followed guidelines for the reporting of neuroimaging meta-analysis [19] (Supplementary Table 1) and was compliant with Preferred Reporting Items for Systematic Reviews and Meta-Analyses (PRISMA) [25] (Supplementary Table 2) and Meta-analysis Of Observational Studies in Epidemiology (MOOSE) guidance [26] (Supplementary Table 3).

### Literature search

PubMed and PsycINFO were searched from inception to 10/03/2021, using a search syntax modified from a previous meta-analysis of cognitive deficits in PUI [12] and adapted for the two databases: (“imaging” OR “MRI” OR “VBM” OR “voxel-based morphometry”) AND (“internet use” OR “internet addiction” OR “smartphone use” OR “smartphone addiction” OR “gaming addiction” OR “internet gaming disorder” OR “PIU” OR “PUI”). This yielded a total of 546 hits in PubMed and 225 hits in PsycINFO, representing 613 unique records after de-duplication.

### Study selection

**Figure 1:**
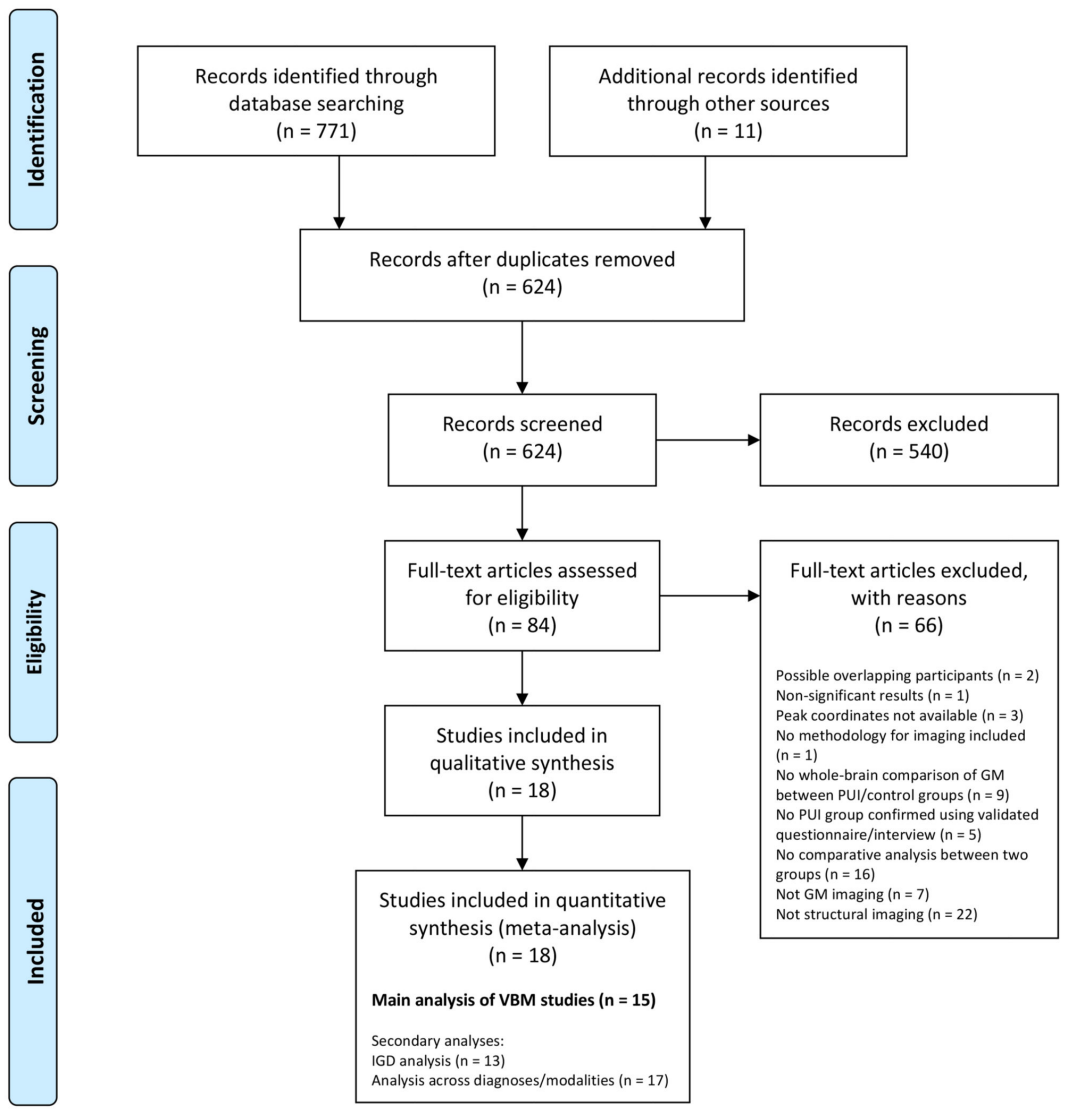
Preferred Reporting Items for Systematic Reviews and Meta-Analyses (PRISMA) flow diagram. Diagram adapted from Moher et al. (2009) [25] using the template available from prisma-statement.org. The references of excluded full text articles are provided in the online supplement (Supplementary Methods 2). GM = gray matter; IGD = Internet gaming disorder; PUI = Problematic Usage of the Internet; VBM = voxel-based morphometry.

Selection took place in two stages, screening and eligibility assessment (Figure 1). In the screening stage, two researchers independently screened titles and abstracts. Studies were excluded if they were clearly out of scope, if they were not peer-reviewed publications or not written in English. Our rationale for excluding non-English studies was that translation of manuscripts into English would have required access to specialist technical imaging expertise in a variety of languages, for which resourcing was not available in this study. All studies deemed relevant by either researcher proceeded to eligibility assessment. Reference lists of all studies proceeding to eligibility assessment and all identified relevant reviews/meta-analyses were hand-searched by one researcher and studies of possible relevance were identified and proceeded to eligibility assessment.

In the eligibility assessment stage, full texts were assessed independently by two researchers. Discrepancies were resolved by consensus discussion. Included studies: (1) were original, cross-sectional comparative studies that used structural gray matter imaging in a PUI population versus a control group, in which PUI was confirmed using a psychiatric interview or a validated questionnaire; (2) reported a whole brain analysis (a requirement for the type of meta-analytic approach we used); and (3) reported peak coordinates for gray matter differences between the PUI and control groups. For studies that satisfied (1) but not (2) or (3), the authors were systematically contacted by email to inquire whether the required information was available. Studies reporting no significant peak coordinates in a whole brain analysis were excluded.

Where there were concerns regarding possible overlapping samples between studies, authors were contacted for further information. In the absence of additional information, the study with the largest sample was included in the relevant meta-analysis.

### Data extraction and study quality

**Figure 2:**
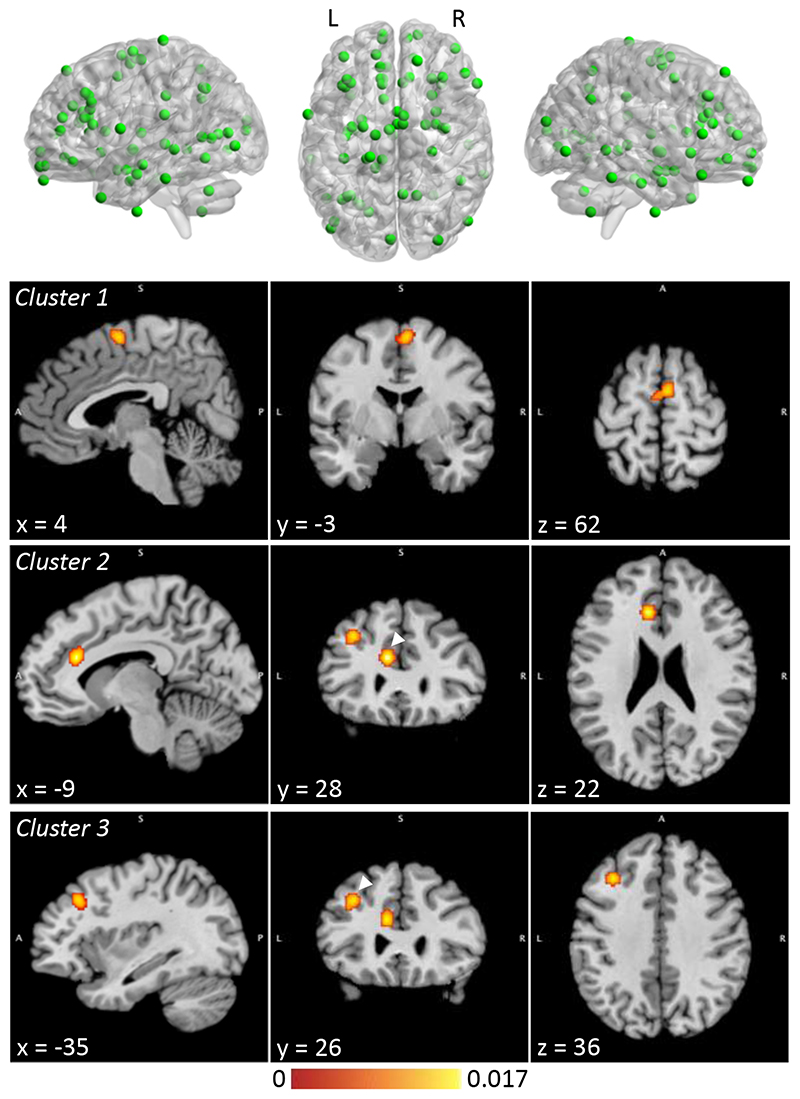
Meta-analysis of voxel-based morphometry studies, showing coordinates included in the analysis and significant clusters. Clusters are shown at their weighted centers. Color bar shows ALE score. Where two clusters are seen in a single image, the relevant cluster is indicated with an arrowhead. ALE = anatomical likelihood estimation; L = left; R = right.

Details regarding data extraction and extracted peak coordinates for each study are provided in the supplementary material (Supplementary Methods 3, Supplementary Table 4). Peak coordinates were visualized with the BrainNet Viewer (http://www.nitrc.org/projects/bnv/) [27] (Figure 2, Supplementary Figure 1).

Some studies reported multiple sets of “experiments” for the same group of subjects, where “experiment” refers to a single contrast producing a set of coordinates [28]. In these cases, results were combined in order that a maximum of two experiments per subject group (increases and decreases in gray matter, i.e. PUI > control and PUI < control) were included in the analysis, consistent with previous studies [28, 29].

To facilitate quality assessment, we designed a study quality checklist, which was adapted from checklists used in previous meta-analyses (Supplementary Methods 3). Relevant information was extracted from studies (Supplementary Table 5) and included in a qualitative literature synthesis.

### Statistical analysis

We carried out coordinate-based meta-analysis using ALE, which is a meta-analytic technique focusing on spatial convergence of foci [22–24]. Using GingerALE Version 3.0.2 (http://www.brainmap.org/ale/), ALE was used to test for significant spatial convergence of foci between experiments, where the null hypothesis was random spatial association [29–31]. ALE first models foci as peaks of three-dimensional Gaussian distributions, to represent the spatial uncertainty of each reported coordinate, taking into account the number of subjects (experiments with fewer subjects have a wider Gaussian) [30]. A “modeled-activation” map for each experiment is created by merging these probability distributions, controlling for the effects of multiple foci being reported close together in a single experiment [29]. ALE scores are then calculated for each voxel by taking the union of the modeled-activation maps and tested against a null distribution with correction for multiple comparisons [31]. Cluster-level family-wise error correction was used, with a cluster-forming threshold on the voxel-level of *P* < .001 and a cluster-level threshold of *P* < .05 [19, 32]. As per the protocol and based on published simulations, at least 17 experiments were required to carry out an ALE meta-analysis, to control for the influence of any individual experiment [32].

Experiments included in each analysis are reported in the supplement (Supplementary Table 6). Significant clusters were visualized using Mango (rii.uthscsa.edu/mango) (Figure 2) and interpreted directionally by inspecting those case-control studies directly contributing to the results.

Where significant clusters were identified, a leave-one-out jackknife sensitivity analysis was performed to assess the robustness of the results; i.e. the meta-analysis was repeated multiple times, leaving out one experiment each time. Results from each iteration were inspected to determine whether significant clusters were preserved (Supplementary Table 7) and, where clusters remained significant, whether their characteristics had altered (Supplementary Table 8).

## Results

From a total of 624 studies considered, 15 were identified for the main analysis focusing on VBM (including 355 individuals with PUI and 363 controls). Secondary analyses were also conducted, (a) focusing on studies examining IGD specifically (rather than PUI in general; 363 individuals with IGD and 373 controls); and (b) adding identified non-VBM studies to the VBM studies (455 individuals with PUI and 466 controls) (Figure 1, Supplementary Table 6). A full list of studies excluded at eligibility assessment, with reasons for exclusion, is provided in the supplementary material (Supplementary Methods 2).

Table 1 summarizes the characteristics of included studies [33–50] and Table 2 shows gray matter regions displaying significant differences between PUI and control groups for each study. Across the whole identified literature, six studies reported the use of a diagnostic interview to confirm PUI (Supplementary Table 5). Mood/anxiety disorders and attention-deficit/hyperactivity disorder (ADHD)/impulsivity were each assessed using validated questionnaires by 11 studies. None of the studies reported the use of a validated questionnaire to assess for impulse control/gambling comorbidities.

The VBM meta-analysis (19 experiments from 15 studies, including 73 foci) identified significant clusters mapping to the medial/superior frontal gyri; the left ACC/cingulate gyrus; and the left middle frontal/precentral gyri (Figure 2, Table 3). The experiments contributing to these clusters all indicated reduced gray matter in PUI participants compared to controls (Table 3). The secondary analyses did not yield significant clusters (Table 3, Supplementary Figure 1).

Leave-one-out jackknife analysis of the VBM meta-analysis demonstrated the following results. The two largest clusters mapping to the medial/superior frontal gyri and the left ACC/cingulate gyrus were each replicated in 16 out of 19 iterations. The smaller cluster mapping to the left middle frontal/precentral gyri was replicated in 15 out of 19 iterations (Supplementary Table 7). Regions and extents of the jackknife findings are shown in Supplementary Table 8.

## Discussion

To our knowledge, this is the first ALE meta-analysis to investigate gray matter structural differences between individuals with PUI and controls. PUI was defined as encompassing problematic behaviors related to the Internet, irrespective of modality or type of activity. Focusing on voxel-based morphometry (VBM) studies, we found significantly reduced gray matter in PUI, versus controls, in the ACC, DLPFC and SMA. These findings broadly confirmed our hypothesis given that these regions are heavily implicated, in other work, with reward processing and top-down inhibitory control [51, 52].

While previous non-ALE meta-analyses identified brain abnormalities in IGD [20, 21], we did not find, in our secondary analysis, significant abnormalities in IGD using ALE. We suspect this may relate to statistical power, as implicated regions in prior IGD work overlap with those herein found to be significantly abnormal in PUI. Also, our secondary analysis pooling all methodologies similarly did not identify significant abnormalities. This could reflect loss of power arising from inclusion of heterogeneous research methodologies. Indeed, there are discrepancies between gray matter volume and cortical thickness measures when applied to the same datasets and so they represent complementary, rather than interchangeable, measures [53, 54].

Meta-analytic confirmation of structural alterations in the ACC, DLPFC and SMA in VBM studies provides insight into the possible neurobiology of PUI, even though structural abnormalities do not necessarily reflect functional abnormalities. The DLPFC and ACC are part of fronto-striatal circuitry that has been implicated in reward processing and inhibitory control [51]; hence, their dysfunction could contribute to elements of repetitive internet-based behavior. In the context of IGD, incongruent response errors in the Stroop task have been found to correlate with ACC structure, suggesting a role for the ACC in cognitive control [55], while ACC and DLPFC structure have also been correlated with impulsivity [33, 50]. The SMA is involved in complex action and has been implicated in task-switching and stop-signal task performance [56]. Identification of structural differences in brain areas related to decision making and inhibitory control supports the Interaction of Person-Affect-Cognition-Execution model, proposed by Brand and colleagues, which suggests that reductions in executive functions and inhibitory control contribute to the development of Internet-use disorders [57].

It is important to consider possible methodological limitations relating to the literature that contributed to the analyses. Control group definitions varied between studies, with some studies recruiting participants reporting minimal Internet use and others recruiting regular Internet users (Table 1). There were also differences in diagnosis and comorbidity screening (Supplementary Table 5). A minority of studies (six out of eighteen) reported using a diagnostic interview to confirm PUI. Less than two thirds reported the use of a formal screening tool assessing mood/anxiety disorders and ADHD/impulsivity, which are known to be associated with PUI [5, 58]. Finally, none of the included studies reported use of a standardized tool for assessing impulse control/gambling such as the Minnesota Impulsive Disorders Interview [59]. Thus, further high quality case-control neuroimaging studies are needed to fully elucidate the contribution of other underlying mental disorders to the neuroimaging findings associated with PUI.

In future, adoption of consensus diagnostic criteria for PUI is expected to reduce heterogeneity. Use of clearly defined control groups, representing both participants with minimal Internet use and participants demonstrating high involvement but non-problematic use, will become important in identifying gray matter changes specific to PUI. This may involve changes to current widely-used criteria, as a Delphi study involving an international expert panel found that some published criteria for gaming disorder were not clinically relevant and some may not distinguish high but non-problematic involvement from problematic involvement in gaming [60]. In addition, the majority of studies included in the current meta-analysis investigated IGD, most participants were male, and most studies were conducted in China and in South Korea. In future, it will be important for studies to investigate a wider spectrum of PUI behaviors in a range of populations. By investigating a range of PUI behaviors and rigorously measuring the contribution of comorbidities, future studies will provide detailed information regarding the similarities and differences in gray matter structure between PUI subtypes and how these relate to brain structural differences identified in other mental disorders.

### Strengths and Limitations

The strengths of this study include the use of an inclusive search strategy to systematically identify relevant studies, coupled with a systematic process to gather unpublished information/data from study authors, and exclusion of potentially duplicated datasets. An important limitation of the current study is that it was not possible to assess the contributions of specific study and sample characteristics to clusters identified in ALE analysis, as each cluster result was primarily driven by a relatively small number of studies. In addition, it was not possible to carry out subgroup meta-analyses to assess, formally, particular directions of gray matter change (i.e., increases and decreases) within ALE itself due to a small number of eligible experiments. Therefore, the current study interpreted directionality by inspecting findings from case-control studies contributing significantly to a given ALE cluster. For resource availability reasons, another limitation is that the study did not include non-English publications. Finally, as our analysis used ALE and the null hypothesis was a lack of spatial convergence across the whole brain, it was not possible to include studies reporting non-significant results or studies using only region-of-interest analysis [19]. Only one study was excluded due to a lack of significant results (Supplementary Methods 2). There have been a number of studies assessing gray matter changes in particular regions-of-interest in PUI, including the striatum [33, 61–68], frontal cortex [38, 61, 69–73], cingulate cortex [38, 63, 66, 67, 71, 73], amygdala [61, 63, 66, 67], insula [73, 74] and temporal cortex [73].

### Conclusions

This meta-analysis of VBM studies confirmed reduced gray matter of the SMA, left ACC and left DLPFC in PUI. Further research is needed to understand the nature of gray matter differences across different PUI behaviors (such as whether they constitute vulnerability markers or stem from PUI itself) and elucidate the contribution of underlying mental health diagnoses, as well as the influence of variation in study and participant characteristics on such findings.

## Supplementary Material

Supplementary information is available at MP’s website.

Supplementary Information

## Figures and Tables

**Table 1:** Characteristics of included studies

Study (first author, year)	Geographic area	Age category	Gender mix	PUI definition	PUI type	Control definition	PUI			Control		
							N	Mean age (SD)	% M	N	Mean age (SD)	% M
Choi et al. (2017) [33]	South Korea	Adults	Male only	Males in their 20s and 30s who mostly played League of Legends, FIFA or Sudden Attack; fulfilled proposed DSM criteria.	IGD	Non-gaming users	22	29.5 (4.7)	100	24	27.2 (4.9)	100
Han et al. (2012) [34]	South Korea	Youth	Male only	YIAT >50; game play time >4 h/day/30 h/week; impaired behaviors or distress	OGA	Healthy comparison group; game play time <3 h/day and <3 day/week	20	20.9 (2.0)	100	18	20.9 (2.1)	100
Horvath et al. (2020) [35]	Germany	Youth	Mixed	Smartphone owners aged 18-30 expressing interest in a study of “dysfunctional smartphone use”; SAS-SV >31 (males), >33 (females)	SPA	Smartphone owners aged 18-30 expressing interest in a study of “dysfunctional smartphone use”; SAS-SV below cut-off	22	22.5 (3.0)	32	26	23.0 (3.2)	31
Jin et al. (2016) [36]	China	Youth	Mixed	Participated in online games such as League of Legends as major online behavior; fulfilled proposed DSM criteria; YIAT >50	IGD	Healthy control group with Internet use; participated in online games such as League of Legends as major online behavior; YIAT 20-30	25	19.1 (1.1)	64	21	18.8 (1.8)	67
Ko et al. (2015) [37]	Taiwan	Youth/ Adults^1^	Male only	IGD diagnosis for >2 years according to DCIA; participated in online gaming for average of ≥4 h/day on weekdays and ≥8 h/day on weekends	IGD	Never fulfilled DCIA	30	23.6 (2.5)	100	30	24.2 (2.5)	100
Lee, Namkoong et al. (2018)^2^ [50]	South Korea	Youth/ Adults^1^	Male only	YIAT >50; reported gaming as the primary purpose of their Internet use; fulfilled proposed DSM criteria	IGD	Healthy controls; YIAT <50; spent <2 hours/day on online gaming	31	24.0 (2.6)	100	30	23.0 (2.8)	100
Lee, Park et al. (2018)^3^ [38]	South Korea	Youth	Male only	YIAT ≥50; reported main use of Internet was playing games; clinician-administered interview to assess the core components of addiction	IGD	Healthy controls	45	23.8 (1.5)	100	35	23.4 (1.7)	100
Lin et al. (2015) [39]	China	Youth	Male only	YIAT ≥50; reported “spending most of their online time playing online games (>50 %)”	IGA	Healthy controls	35	22.2 (3.1)	100	36	22.3 (2.5)	100
Seok and Sohn (2018) [40]	South Korea	Youth	Male only	Fulfilled proposed DSM criteria	IGD	Healthy controls	20	21.7 (2.7)	100	20	22.4 (2.6)	100
Sun et al. (2014) [41]	China	Youth	Mixed	Fulfilled modified YDQ criteria; subjects characterised as the IGA subtype (mostly focused on online gaming when using the Internet)	IGA	Healthy controls; sometimes played online/mobile games but did not meet diagnostic criteria for IGA^4^	18	20.5 (3.6)	83	21	22.0 (2.4)	86
C. Wang et al. (2021) [42]	China	Youth	Mixed	YIAT ≥50; fulfilled ≥5 proposed DSM criteria	IGD	Healthy controls; YIAT <50; fulfilled <5 proposed DSM criteria; never played online games or spent <2 hours/day playing online games in the last 2 years^4^	26	23.2 (2.5)	54	28	23.4 (2.8)	54
S. Wang et al. (2018)^3^ [43]	China	Youth	Male only	Fulfilled ≥5 proposed DSM criteria; YIAT ≥50; reported Internet gaming as primary online activity	IGD	Normal controls; fulfilled <4 proposed DSM criteria; YIAT <30	48	20.6 (1.0)	100	32	21.1 (2.2)	100
Y. Wang et al. (2016) [44]	China	Youth	Mixed	MPAI >51	MPD	Non-MPD	34	21.6 (2.1)	38	34	21.7 (1.9)	38
Z. Wang et al. (2018)^3^ [45]	China	Youth	Mixed	Regularly played “League of Legends” for at least a year; fulfilled ≥5 proposed DSM criteria; YIAT ≥50	IGD	Recreational game users; regularly played “League of Legends” for at least a year and as frequently as the IGD subjects (at least 5/7 days and >14h/week); fulfilled <4 proposed DSM criteria; YIAT <50	38	20.7 (2.1)	71	66	21.3 (2.0)	56
Weng et al. (2013) [46]	China	Youth	Mixed	Fulfilled modified YDQ criteria; playing online games was primary Internet activity	OGA	Healthy individuals without OGA	17	16.3 (3.0)	24	17	15.5 (3.2)	12
Yoon et al. (2017) [47]	South Korea	Youth/ Adults^1^	Male only	Fulfilled proposed DSM criteria; YIAT ≥50; spent >4h/day and >30h/week involved in Internet gaming	IGD	Healthy controls; used the Internet <2 h/day	19	22.9 (5.2)	100	25	25.4 (3.8)	100
Yuan et al. (2011) [48]	China	Youth	Mixed	Fulfilled modified YDQ criteria	IAD	Healthy controls; spent <2 h/day on the internet	18	19.4 (3.1)	67	18	19.5 (2.8)	67
Zhou et al. (2011) [49]	China	Youth	Mixed	Fulfilled modified YDQ criteria	IA	Healthy individuals, sometimes played games but did not meet diagnostic criteria for IA^4^	18	17.2 (2.6)	89	15	17.8 (2.6)	87

1 Mean ages reported in the two samples fell into both the Youth and Adults categories.

2 Included in the voxel-based morphometry meta-analysis but not in secondary analyses.

3 Surface-based morphometry studies included only in secondary analyses.

4 Includes information from unpublished sources.

DCIA = diagnostic criteria for Internet addiction; DSM = Diagnostic and Statistical Manual of Mental Disorders (5^th^ ed.); IA = Internet addiction; IAD = Internet addiction disorder; IGA = Internet gaming addiction; IGD = Internet gaming disorder; M = male; MPAI = mobile phone addiction index; MPD = mobile phone dependence; OGA = online game addiction; PUI = Problematic Usage of the Internet; SAS-SV = short version of the Smartphone Addiction Scale; SPA = smartphone addiction; YDQ = Young’s diagnostic questionnaire; YIAT = Young’s online Internet addiction test.

**Table 2:** Gray matter regions with significant differences between PUI and control groups in included studies

Study	Anatomy measure	Gray matter regions with significant differences between groups	
		PUI < control	PUI > control
Choi et al. (2017) [33]	GMD	L DLPFC	-
Han et al. (2012) [34]	GMV	B inferior temporal gyrus, R middle occipital gyrus, L inferior occipital gyrus, L fusiform gyrus	L thalamus, L angular gyrus
Horvath et al. (2020) [35]	GMV	L anterior insula, L inferior temporal cortex, L parahippocampal cortex	L supramarginal gyrus
Jin et al. (2016) [36]	GMV	B DLPFC, B OFC, B ACC, R SMA	-
Ko et al. (2015) [37]	GMD	B amygdala	-
Lee, Namkoong et al. (2018)^1^ [50]	GMV	R ACC, R SMA, L ventrolateral prefrontal cortex, L inferior parietal lobule, L anterior temporal lobe	-
Lee, Park et al. (2018)^2^ [38]	CTh	R SMA, L frontal eye field, L posterior cingulate cortex, L superior parietal lobule	-
Lin et al. (2015) [39]	GMD	B inferior frontal gyrus, L insula, R precuneus, L cingulate gyrus, R hippocampus	-
Seok and Sohn (2018) [40]	GMV	B middle frontal gyrus	L caudate
Sun et al. (2014) [41]	GMV	L precentral gyrus	R inferior temporal gyrus, R middle temporal gyrus, R parahippocampal gyrus
C. Wang et al. (2021) [42]	GMV	L superior frontal gyrus, L SMA	-
S. Wang et al. (2018)^2^ [43]	CTh	B banks of superior temporal sulcus, R precuneus, R precentral gyrus, R inferior parietal cortex, L middle temporal gyrus	B insula, R inferior temporal gyrus
Y. Wang et al. (2016) [44]	GMV	R superior frontal gyrus, R inferior frontal gyrus, B medial frontal gyrus, R middle occipital gyrus, L ACC, B thalamus	-
Z. Wang et al. (2018)^2^ [45]	CTh and CV	L inferior parietal lobule, L postcentral gyrus, L precentral gyrus, L lateral OFC, B cuneus, R middle temporal gyrus, B superior parietal lobule, R lateral occipital cortex, L superior temporal gyrus, R supramarginal gyrus, R banks of superior temporal sulcus	R isthmus of cingulate gyrus
Weng et al. (2013) [46]	GMV	B insula, R OFC, R SMA	-
Yoon et al. (2017) [47]	GMV^3^	-	B hippocampus/amygdala, R precuneus
Yuan et al. (2011) [48]	GMV	B DLPFC, B SMA, B OFC, B cerebellum, L rostral ACC	-
Zhou et al. (2011) [49]	GMD	L ACC, L posterior cingulate cortex, L insula, L lingulate gyrus	-

1 Included in the voxel-based morphometry meta-analysis but not in secondary analyses.

2 Surface-based morphometry studies included only in secondary analyses.

3 CTh was also investigated but there were no significant differences in CTh between individuals with PUI and other groups.

ACC = anterior cingulate cortex; B = bilateral; CTh = cortical thickness; CV = cortical volume; DLPFC = dorsolateral prefrontal cortex; GMD = gray matter density; GMV = gray matter volume; L = left; OFC = orbitofrontal cortex; PUI = Problematic Usage of the Internet; R = right; SMA = supplementary motor area.

**Table 3:** Characteristics of significant clusters identified by ALE meta-analysis

Cluster	Volume (mm^3^)	Peak MNI coordinates			ALE score	*P* value	Hemisphere	Gyrus	BA	Contributing experiments
		X	Y	Z						
**VBM meta-analysis**										
1	776	6	-2	62	.014	9.67 x10^−6^	79% R, 21% L	98% medial frontal gyrus, 2% superior frontal gyrus	100% BA6	Jin et al. (2016), IGD<C [36]; Weng et al. (2013), OGA<C [46]; Yuan et al. (2011), IAD<C [48]
		-2	-6	62	.009	2.08 x10^−4^				
2	760	-10	28	20	.017	3.94 x10^−7^	100% L	71% anterior cingulate, 29% cingulate gyrus	67% BA32, 33% BA24	Jin et al. (2016), IGD<C [36]; Yuan et al. (2011), IAD<C [48]; Zhou et al. (2011), IA<C [49]
3	656	-34	26	36	.015	2.88 x10^−6^	100% L	58% middle frontal gyrus, 42% precentral gyrus	67% BA9, 33% BA8	Choi et al. (2017), IGD<C [33]; Jin et al. (2016), IGD<C [36]; Yuan et al. (2011), IAD<C [48]
**IGD meta-analysis**										
No significant clusters										
**Meta-analysis across diagnoses/modalities**										
No significant clusters										

ALE = anatomical likelihood estimation; BA = Brodmann Area; C = control; IA = Internet addiction; IAD = Internet addiction disorder; IGD = Internet gaming disorder; L = left; MNI = Montreal Neurological Institute; OGA = online game addiction; R = right; VBM = voxel-based morphometry.
