# Supplementary material for: Structural gray matter differences in Problematic Usage of the Internet: a systematic review and meta-analysis
Source: Mol Psychiatry. Author manuscript; Available in PMC 2022 May 2. (PMC9054652; doi:10.1038/s41380-021-01315-7)
Supplement: Supplementary Information [file EMS133024-supplement-Supplementary_Information.docx]

**Structural gray matter differences in Problematic Usage of the Internet: a systematic review and meta-analysis**

Jeremy E. Solly, Roxanne W. Hook, Jon E. Grant, Samuele Cortese and Samuel R. Chamberlain

**Supplementary Information**

[Supplementary Figure 1: Coordinates included in the secondary analyses 2](#_Toc75260306)

[Supplementary Methods 1: Changes compared to the pre-registered protocol 3](#_Toc75260307)

[Supplementary Methods 2: Studies excluded in the eligibility assessment stage with reasons for exclusion 4](#_Toc75260308)

[Supplementary Methods 3: Details regarding data extraction and study quality information 7](#_Toc75260309)

[Supplementary Table 1: Checklist for neuroimaging meta-analyses 8](#_Toc75260310)

[Supplementary Table 2: Preferred Reporting Items for Systematic Reviews and Meta-Analyses (PRISMA) checklist 10](#_Toc75260311)

[Supplementary Table 3: Reporting checklist for meta-analyses of observational studies 12](#_Toc75260312)

[Supplementary Table 4: Extracted peak coordinates for each study 14](#_Toc75260313)

[Supplementary Table 5: Study quality checklist 17](#_Toc75260314)

[Supplementary Table 6: Experiments included in each meta-analysis 18](#_Toc75260315)

[Supplementary Table 7: Jackknife sensitivity analysis of the three clusters identified in the voxel-based morphometry meta-analysis 19](#_Toc75260316)

[Supplementary Table 8: Changes in cluster characteristics during jackknife sensitivity analysis 20](#_Toc75260317)

[References 21](#_Toc75260318)

# Supplementary Figure 1: Coordinates included in the secondary analyses


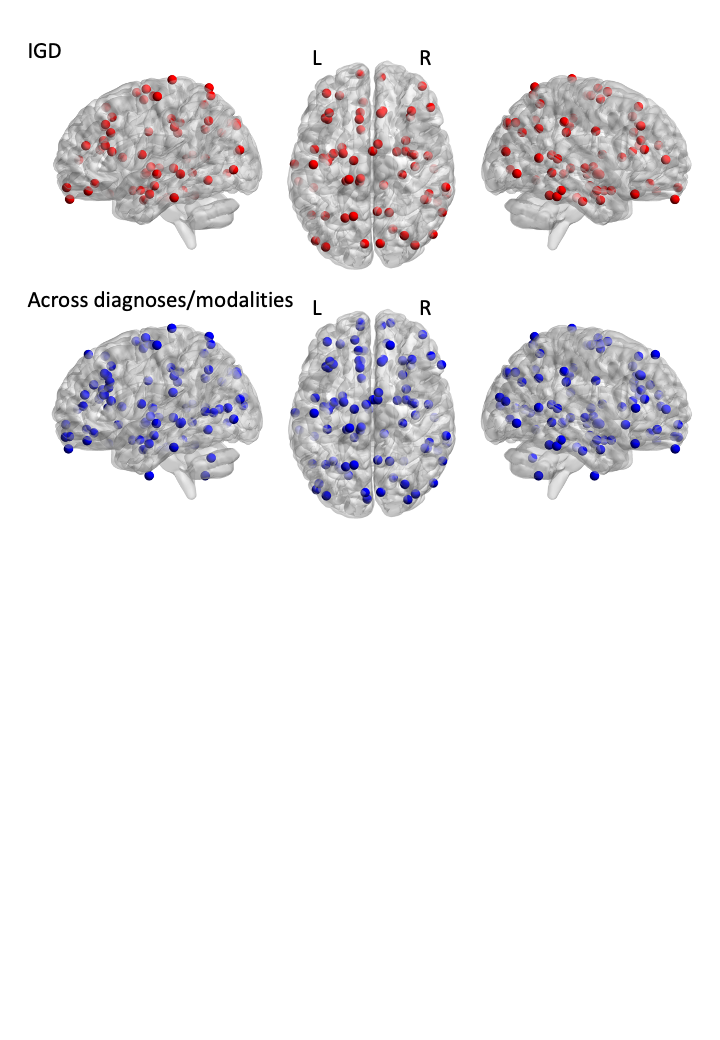


Coordinates included in the IGD meta-analysis and the meta-analysis across diagnoses/modalities. These datasets did not produce significant clusters in ALE meta-analysis.

ALE = anatomical likelihood estimation; IGD = Internet gaming disorder; L = left; R = right.

# Supplementary Methods 1: Changes compared to the pre-registered protocol

The protocol for this study was pre-registered in the international prospective register of systematic reviews (PROSPERO 2020 CRD42020176234). Changes are as follows:

- The protocol stated that the literature search would be carried out up to 23/03/2020. An updated search to 15/12/2020 was carried out prior to data extraction and an updated search to 10/03/2021 was carried out during manuscript preparation.
- We stated that included studies would be: “*original, cross-sectional comparative studies that include structural grey matter imaging in an internet addiction population versus a healthy comparison (control group).*” This statement made no restriction on study image analysis methodology. We included voxel-based morphometry (VBM) and surface-based morphometry data, but excluded data from diffusional kurtosis imaging, as only one identified study used this method [1].
- We stated that: “*The quality assurance control will be performed independently by two psychiatrists, who will then meet together to arrive at a consensus. All papers in scope will be assessed against the quality standard individually and will receive a score between 0 and 10.*” The quality assurance control was performed independently by two researchers (one psychiatrist and one medical doctor) and all papers received a score between 0 and 13.
- We stated that: “*Two researchers will perform initial screening. Full text screening for final inclusion of studies will be carried out by consensus between three researchers.*” Full text screening was carried out by consensus between two researchers. Consensus discussion included senior researchers when required.
- We stated that: “*We will perform subgroup analysis for any specific internet use disorder (e.g. IGD) for which there are sufficient data.*” We performed a primary analysis focusing on all VBM studies. In addition, we conducted two secondary analyses, focusing on (a) IGD studies specifically; and (b) addition of non-VBM studies. This was not specified in the protocol because it was not possible to identify modalities with a sufficient number of studies until after the literature search had been conducted.
- Where significant clusters were identified, we carried out a leave-one-out jackknife analysis to assess the robustness of the results.

# Supplementary Methods 2: Studies excluded in the eligibility assessment stage with reasons for exclusion

**Possible overlapping participants with included studies**

1. Lee D, Namkoong K, Lee J, Jung YC. Dorsal striatal functional connectivity changes in internet gaming disorder: a longitudinal magnetic resonance imaging study. *Addict Biol*. 2021;26(1):e12868.
2. Lee D, Namkoong K, Lee J, Jung YC. Preliminary evidence of altered gray matter volume in subjects with internet gaming disorder: associations with history of childhood attention-deficit/hyperactivity disorder symptoms. *Brain Imaging Behav*. 2019;13(3):660-668.

**Non-significant results in whole brain analysis**

1. Lee D, Namkoong K, Lee J, Lee BO, Jung YC. Lateral orbitofrontal gray matter abnormalities in subjects with problematic smartphone use. *J Behav Addict*. 2019;8(3):404-411.

**Peak coordinates not available**

1. Hong SB, Kim JW, Choi EJ, et al. Reduced orbitofrontal cortical thickness in male adolescents with internet addiction. *Behav Brain Funct*. 2013;9:11.
2. Wang H, Jin C, Yuan K, et al. The alteration of gray matter volume and cognitive control in adolescents with internet gaming disorder. *Front Behav Neurosci*. 2015;9:64.
3. Yuan K, Cheng P, Dong T, et al. Cortical thickness abnormalities in late adolescence with online gaming addiction. *PLoS One*. 2013;8(1):e53055.

**No methodology for imaging included**

1. Lee D, Hong SJ, Jung YC, Park J, Kim IY, Namkoong K. Altered heart rate variability during gaming in internet gaming disorder. *Cyberpsychol Behav Soc Netw*. 2018;21(4):259-267.

**No whole brain comparison of gray matter between Problematic Usage of the Internet (PUI) and control groups**

1. Cai C, Yuan K, Yin J, et al. Striatum morphometry is associated with cognitive control deficits and symptom severity in internet gaming disorder. *Brain Imaging Behav*. 2016;10(1):12-20.
2. Du X, Qi X, Yang Y, et al. Altered structural correlates of impulsivity in adolescents with internet gaming disorder. *Front Hum Neurosci*. 2016;10:4.
3. Liu L, Yip SW, Zhang JT, et al. Activation of the ventral and dorsal striatum during cue reactivity in internet gaming disorder. *Addict Biol*. 2017;22(3):791-801.
4. Pan N, Yang Y, Du X, et al. Brain structures associated with internet addiction tendency in adolescent online game players. *Front Psychiatry*. 2018;9:67.
5. Park CH, Chun JW, Cho H, Kim DJ. Discriminating pathological and non-pathological internet gamers using sparse neuroanatomical features. *Front Psychiatry*. 2018;9:291.
6. Schmidt C, Morris LS, Kvamme TL, Hall P, Birchard T, Voon V. Compulsive sexual behavior: prefrontal and limbic volume and interactions. *Hum Brain Mapp*. 2017;38(3):1182-1190.
7. Wang Z, Hu Y, Zheng H, Yuan K, Du X, Dong G. Females are more vulnerable to internet gaming disorder than males: evidence from cortical thickness abnormalities. *Psychiatry Res Neuroimaging*. 2019;283:145-153.
8. Yuan K, Yu D, Cai C, et al. Frontostriatal circuits, resting state functional connectivity and cognitive control in internet gaming disorder. *Addict Biol*. 2017;22(3):813-822.
9. Zsidó AN, Darnai G, Inhóf O, et al. Differentiation between young adult internet addicts, smokers, and healthy controls by the interaction between impulsivity and temporal lobe thickness. *J Behav Addict*. 2019;8(1):35-47.

**No PUI group confirmed using psychiatric interview/validated questionnaire**

1. He Q, Turel O, Brevers D, Bechara A. Excess social media use in normal populations is associated with amygdala-striatal but not with prefrontal morphology. *Psychiatry Res Neuroimaging*. 2017;269:31-35.
2. He Q, Turel O, Wei L, Bechara A. Structural brain differences associated with extensive massively-multiplayer video gaming. *Brain Imaging Behav*. 2021;15(1):364-374.
3. Kühn S, Romanowski A, Schilling C, et al. The neural basis of video gaming. *Transl Psychiatry*. 2011;1(11):e53.
4. Mohammadi B, Szycik GR, Te Wildt B, Heldmann M, Samii A, Münte TF. Structural brain changes in young males addicted to video-gaming. *Brain Cogn*. 2020;139:105518.
5. Zhou F, Montag C, Sariyska R, et al. Orbitofrontal gray matter deficits as marker of internet gaming disorder: converging evidence from a cross-sectional and prospective longitudinal design. *Addict Biol*. 2019;24(1):100-109.

**No comparative analysis between two groups**

1. Altbäcker A, Plózer E, Darnai G, et al. Problematic internet use is associated with structural alterations in the brain reward system in females. *Brain Imaging Behav*. 2016;10(4):953-959.
2. He Q, Turel O, Bechara A. Brain anatomy alterations associated with social networking site (SNS) addiction. *Sci Rep*. 2017;7:45064.
3. Inhóf O, Zsidó AN, Perlaki G, et al. Internet addiction associated with right pars opercularis in females. J Behav Addict. 2019;8(1):162-168.
4. Kühn S, Gallinat J. Amount of lifetime video gaming is positively associated with entorhinal, hippocampal and occipital volume. *Mol Psychiatry*. 2014;19(7):842-847.
5. Kühn S, Gallinat J. Brains online: structural and functional correlates of habitual internet use. *Addict Biol*. 2015;20(2):415-422.
6. Kühn S, Gallinat J. Brain structure and functional connectivity associated with pornography consumption: the brain on porn. *JAMA Psychiatry*. 2014;71(7):827-834.
7. Kühn S, Lorenz R, Banaschewski T, et al. Positive association of video game playing with left frontal cortical thickness in adolescents. *PLoS One*. 2014;9(3):e91506.
8. Li W, Li Y, Yang W, et al. Brain structures and functional connectivity associated with individual differences in internet tendency in healthy young adults. *Neuropsychologia*. 2015;70:134-144.
9. Montag C, Markowetz A, Blaszkiewicz K, et al. Facebook usage on smartphones and gray matter volume of the nucleus accumbens. *Behav Brain Res*. 2017;329:221-228.
10. Montag C, Zhao Z, Sindermann C, et al. Internet communication disorder and the structure of the human brain: initial insights on WeChat addiction. *Sci Rep*. 2018;8(1):2155.
11. Takeuchi H, Taki Y, Asano K, et al. Impact of frequency of internet use on development of brain structures and verbal intelligence: longitudinal analyses. *Hum Brain Mapp*. 2018;39(11):4471-4479.
12. Takeuchi H, Taki Y, Hashizume H, et al. Impact of videogame play on the brain’s microstructural properties: cross-sectional and longitudinal analyses. *Mol Psychiatry*. 2016;21(12):1781-1789.
13. Turel O, He Q, Brevers D, Bechara A. Delay discounting mediates the association between posterior insular cortex volume and social media addiction symptoms. *Cogn Affect Behav Neurosci*. 2018;18(4):694-704.
14. Zhang M, Bian Y. An analysis of the brain structures underlying the link between pathological internet use and anxiety. *Addict Behav*. 2021;112:106632.
15. Zhou X, Wu R, Liu C, et al. Higher levels of (internet) gaming disorder symptoms according to the WHO and APA frameworks associate with lower striatal volume. *J Behav Addict*. 2020;9(3):598-605.
16. Zou L, Wu X, Tao S, et al. Anterior cingulate gyrus acts as a moderator of the relationship between problematic mobile phone use and depressive symptoms in college students. *Soc Cogn Affect Neurosci*. 2021. Online ahead of print. doi:10.1093/scan/nsab016

**Not gray matter imaging**

1. Cheng H, Liu J. Alterations in amygdala connectivity in internet addiction disorder. *Sci Rep*. 2020;10(1):2370.
2. Dong G, DeVito E, Huang J, Du X. Diffusion tensor imaging reveals thalamus and posterior cingulate cortex abnormalities in internet gaming addicts. *J Psychiatr Res*. 2012;46(9):1212-1216.
3. Dong G, Wu L, Wang Z, Wang Y, Du X, Potenza MN. Diffusion-weighted MRI measures suggest increased white-matter integrity in internet gaming disorder: evidence from the comparison with recreational internet game users. *Addict Behav*. 2018;81:32-38.
4. He Q, Turel O, Bechara A. Association of excessive social media use with abnormal white matter integrity of the corpus callosum. *Psychiatry Res Neuroimaging*. 2018;278:42-47.
5. Kim M, Kim D, Bae S, Han DH, Jeong B. Aberrant structural network of comorbid attention deficit/hyperactivity disorder is associated with addiction severity in internet gaming disorder. *Neuroimage Clin*. 2020;27:102263.
6. Park CH, Chun JW, Cho H, Kim DJ. Alterations in the connection topology of brain structural networks in internet gaming addiction. *Sci Rep*. 2018;8(1):15117.
7. Tymofiyeva O, Yuan JP, Kidambi R, et al. Neural correlates of smartphone dependence in adolescents. *Front Hum Neurosci*. 2020;14:564629.

**Not structural brain imaging**

1. Chen J, Li X, Zhang Q, et al. Impulsivity and response inhibition related brain networks in adolescents with internet gaming disorder: a preliminary study utilizing resting-state fMRI. *Front Psychiatry*. 2021;11:618319.
2. Dong G, Huang J, Du X. Alterations in regional homogeneity of resting-state brain activity in internet gaming addicts. *Behav Brain Funct*. 2012;8:41.
3. Dong G, Lin X, Potenza MN. Decreased functional connectivity in an executive control network is related to impaired executive function in internet gaming disorder. *Prog Neuropsychopharmacol Biol Psychiatry*. 2015;57:76-85.
4. Dong G, Liu X, Zheng H, Du X, Potenza MN. Brain response features during forced break could predict subsequent recovery in internet gaming disorder: a longitudinal study. *J Psychiatr Res*. 2019;113:17-26.
5. Dong G, Wang L, Du X, Potenza MN. Gaming increases craving to gaming-related stimuli in individuals with internet gaming disorder. *Biol Psychiatry Cogn Neurosci Neuroimaging*. 2017;2(5):404-412.
6. Dong G, Wang M, Liu X, Liang Q, Du X, Potenza MN. Cue-elicited craving-related lentiform activation during gaming deprivation is associated with the emergence of Internet gaming disorder. *Addict Biol*. 2020;25(1):e12713.
7. Feng Q, Chen X, Sun J, et al. Voxel-level comparison of arterial spin-labeled perfusion magnetic resonance imaging in adolescents with internet gaming addiction. *Behav Brain Funct*. 2013;9(1):33.
8. Han DH, Kim SM, Bae S, Renshaw PF, Anderson JS. A failure of suppression within the default mode network in depressed adolescents with compulsive internet game play. *J Affect Disord*. 2016;194:57-64.
9. Han X, Wu X, Wang Y, et al. Alterations of resting-state static and dynamic functional connectivity of the dorsolateral prefrontal cortex in subjects with internet gaming disorder. *Front Hum Neurosci*. 2018;12:41.
10. Hong SB, Harrison BJ, Dandash O, et al. A selective involvement of putamen functional connectivity in youth with internet gaming disorder. *Brain Res*. 2015;1602:85-95.
11. Kim YR, Son JW, Lee SI, et al. Abnormal brain activation of adolescent internet addict in a ball-throwing animation task: possible neural correlates of disembodiment revealed by fMRI. *Prog Neuropsychopharmacol Biol Psychiatry*. 2012;39(1):88-95.
12. Ko CH, Hsieh TJ, Chen CY, et al. Altered brain activation during response inhibition and error processing in subjects with internet gaming disorder: a functional magnetic imaging study. *Eur Arch Psychiatry Clin Neurosci*. 2014;264(8):661-672.
13. Lin X, Jia X, Zang YF, Dong G. Frequency-dependent changes in the amplitude of low-frequency fluctuations in internet gaming disorder. *Front Psychol*. 2015;6:1471.
14. Park CH, Chun JW, Cho H, Jung YC, Choi J, Kim DJ. Is the internet gaming-addicted brain close to be in a pathological state? *Addict Biol*. 2017;22(1):196-205.
15. Seo HS, Jeong EK, Choi S, Kwon Y, Park HJ, Kim I. Changes of neurotransmitters in youth with internet and smartphone addiction: a comparison with healthy controls and changes after cognitive behavioral therapy. *AJNR Am J Neuroradiol*. 2020;41(7):1293-1301.
16. Turel O, He Q, Wei L, Bechara A. The role of the insula in internet gaming disorder. *Addict Biol*. 2021;26(2):e12894.
17. Wang L, Wu L, Lin X, et al. Altered brain functional networks in people with internet gaming disorder: evidence from resting-state fMRI. *Psychiatry Res Neuroimaging*. 2016;254:156-163.
18. Wang Y, Zheng L, Wang C, Guo X. Attenuated self-serving bias in people with internet gaming disorder is related to altered neural activity in subcortical-cortical midline structures. *BMC Psychiatry*. 2020;20(1):512.
19. Wang Z, Dong H, Du X, Zhang JT, Dong GH. Decreased effective connection from the parahippocampal gyrus to the prefrontal cortex in Internet gaming disorder: a MVPA and spDCM study. *J Behav Addict*. 2020;9(1):105-115.
20. Yuan K, Qin W, Yu D, et al. Core brain networks interactions and cognitive control in internet gaming disorder individuals in late adolescence/early adulthood. *Brain Struct Funct*. 2016;221(3):1427-1442.
21. Zhang J, Hu Y, Li H, et al. Altered brain activities associated with cue reactivity during forced break in subjects with Internet gaming disorder. *Addict Behav*. 2020;102:106203.
22. Zhang JT, Ma SS, Yip SW, et al. Decreased functional connectivity between ventral tegmental area and nucleus accumbens in internet gaming disorder: evidence from resting state functional magnetic resonance imaging. *Behav Brain Funct*. 2015;11(1):37.

# Supplementary Methods 3: Details regarding data extraction and study quality information

**Data extraction**

Data extraction methods were adapted from a previous meta-analysis of cognitive deficits in Problematic Usage of the Internet (PUI) [2]. Data were extracted from the original papers and recorded in a spreadsheet. Descriptive data included: (a) a geographical determinant in which data collection occurred; (b) key participant demographics (sample size; mean age; age as categorized by mean age: children 0-12, youth 12-24, adults 24-55, older people ≥55; gender distribution in the sample reported as percentage of participants who were male and female; gender distribution categorized as “male only”, “female only” or “mixed”); (c) operationalization of PUI including instrument and cut-off used; (d) criteria for recruitment to the PUI and control groups; (e) measure of gray matter structure assessed; and (f) gray matter regions demonstrating significant differences between the PUI and control groups, including the direction of change. Where a study compared a PUI group to two or more other groups (e.g., professional Internet gamers and non-gamers, or recreational Internet gamers and non-gamers), by preference we used the data for the group most likely to represent healthy controls. Descriptive data were extracted by one researcher and independently checked for accuracy by a second researcher.

Data required for the meta-analysis were extracted independently by two researchers and organized by experiment. Data included: (a) the experiments generating significant results; (b) peak coordinates for the gray matter differences between the PUI and control groups; (c) the standard space in which coordinates were reported, Montreal Neurological Institute (MNI) [3] or Talairach [4]; and (d) the sample size (of either the control or PUI group, whichever was smaller). GingerALE Version 3.0.2 (http://www.brainmap.org/ale/) [5–7] was used to convert coordinates from Talairach to MNI space prior to meta-analysis.

**Study quality**

In addition to the descriptive data extraction outlined above, information relevant to study quality was extracted by one researcher and independently checked for accuracy by a second researcher. The study quality checklist was adapted from checklists used in previous meta-analyses. Aspects relating to PUI research were assessed by adapting the checklist used by Ioannidis et al. (2019) [2] and aspects relating to neuroimaging were assessed by adapting checklists used by Du et al. (2014) [8] and Shepherd et al. (2012) [9]. The final checklist contained 13 items, each of which were scored as present/absent independently by two researchers based on whether they were reported for each study. The checklist items included:

1. Use of a validated screening tool for PUI
2. Use of a diagnostic interview to confirm a diagnosis of PUI
3. Assessment of comorbidities including mood/anxiety disorders using a validated questionnaire (including a report of the questionnaire used)
4. Assessment of substance use comorbidities
5. Assessment of impulse control/gambling comorbidities using a validated questionnaire (including a report of the questionnaire used)
6. Assessment of attention-deficit/hyperactivity disorder or impulsivity using a validated questionnaire (including a report of the questionnaire used)
7. IQ
8. Level of education
9. Sample size >10 per group
10. Magnet strength ≥1.5 T
11. MRI slice thickness ≤3 mm
12. Coordinates in a standard space
13. Statistical parameters for significance

Quality scoring for each study is reported in Supplementary Table 5. In cases where relevant information was absent from a manuscript (e.g., no detailed information provided regarding screening for comorbidities), the authors were contacted for additional information.

# Supplementary Table 1: Checklist for neuroimaging meta-analyses

| The research question is specifically defined | Aim: to identify GM structural differences between subjects with PUI and controls. This used only structural MRI contrasts. |
| --- | --- |
| The literature search was systematic | PubMed and PsycINFO were searched from inception to 10/03/2021, using the following search string:  (“imaging” OR “MRI” OR “VBM” OR “voxel-based morphometry”) AND (“internet use” OR “internet addiction” OR “smartphone use” OR “smartphone addiction” OR “gaming addiction” OR “internet gaming disorder” OR “PIU” OR “PUI”) |
| Detailed inclusion and exclusion criteria are included | Inclusion criteria:   1. Original, cross-sectional comparative studies that included structural GM imaging in a PUI population versus a control group, in which PUI was confirmed using a psychiatric interview or validated questionnaire 2. Report whole brain analysis 3. Report peak coordinates for GM differences between PUI and control groups   Exclusion criteria:   1. Sample overlapping with another included study 2. No significant peak coordinates 3. Not peer-reviewed 4. Not written in English |
| Sample overlap was taken into account | For studies where there were concerns regarding overlapping samples and no additional information, only the study with the largest sample was included in the relevant meta-analysis.  Where multiple experiments were reported, results were combined in order that a maximum of two experiments per subject group (increases and decreases in GM) were included. |
| All experiments use the same search coverage (state how brain coverage is assessed and how small volume corrections and conjunctions are taken into account) | Whole brain analysis only. Studies reporting only carrying out ROI analysis without a whole brain analysis were excluded. |
| Studies are converted to a common reference space | Talairach coordinates were converted to MNI space using GingerALE 3.0.2. |
| Data extraction has been conducted by two investigators (ideal case) or double checked by the same investigator (state how double-checking was performed) | Two investigators (JES and RWH) independently checked inclusion/exclusion criteria.  Two investigators (JES and RWH) independently extracted coordinates.  JES extracted descriptive information, which was double-checked by RWH. |
| The paper includes a table with at least the references, basic study description (e.g. for fMRI tasks: stimuli), contrasts and basic sample descriptions (e.g. size, mean age and gender distribution, specific characteristics) of the included studies, source of information (e.g. contact with authors), reference space | This information is provided in Table 1 and Supplementary Table 4. |
| The study protocol was previously registered and all analyses planned beforehand, including the methods and parameters used for inference, correction for multiple testing, etc. | The meta-analysis was registered at: PROSPERO 2020 CRD42020176234.  We performed a primary analysis focusing on all VBM studies. In addition, we conducted two secondary analyses, focusing on (a) IGD studies specifically; and (b) addition of non-VBM studies. This was not specified in the protocol because it was not possible to identify modalities with a sufficient number of studies until after the literature search had been conducted.  Meta-analysis used the recommended parameters of GingerALE. The less conservative (larger) mask size was used to reduce the number of coordinates located outside the mask. |
| The meta-analysis includes diagnostics | Experiments contributing to each cluster are reported in Table 3. Leave-one-out jackknife analysis is reported in Supplementary Tables 7 and 8. |

Table adapted from Müller et al. (2018) [10], following their recommendation that all authors of neuroimaging meta-analyses complete this checklist.

GM = gray matter; IGD = Internet gaming disorder; MNI = Montreal Neurological Institute; MRI = magnetic resonance imaging; PUI = Problematic Usage of the Internet; ROI = region-of-interest; VBM = voxel-based morphometry.

# Supplementary Table 2: Preferred Reporting Items for Systematic Reviews and Meta-Analyses (PRISMA) checklist

| **Section/Topic** | **#** | **Checklist Item** | **Reported** |
| --- | --- | --- | --- |
| **TITLE** | | | |
| Title | 1 | Identify the report as a systematic review, meta-analysis, or both. | Title |
| **ABSTRACT** | | | |
| Structured summary | 2 | Provide a structured summary including, as applicable: background; objectives; data sources; study eligibility criteria, participants, and interventions; study appraisal and synthesis methods; results; limitations; conclusions and implications of key findings; systematic review registration number. | Abstract. Registration number provided in Methods. |
| **INTRODUCTION** | | | |
| Rationale | 3 | Describe the rationale for the review in the context of what is already known. | Introduction |
| Objectives | 4 | Provide an explicit statement of questions being addressed with reference to participants, interventions, comparisons, outcomes, and study design (PICOS). | Introduction and Methods |
| **METHODS** | | | |
| Protocol and registration | 5 | Indicate if a review protocol exists, if and where it can be accessed (e.g., Web address), and, if available, provide registration information including registration number. | Methods |
| Eligibility criteria | 6 | Specify study characteristics (e.g., PICOS, length of follow-up) and report characteristics (e.g., years considered, language, publication status) used as criteria for eligibility, giving rationale. | Methods |
| Information sources | 7 | Describe all information sources (e.g., databases with dates of coverage, contact with study authors to identify additional studies) in the search and date last searched. | Methods |
| Search | 8 | Present full electronic search strategy for at least one database, including any limits used, such that it could be repeated. | Methods |
| Study selection | 9 | State the process for selecting studies (i.e., screening, eligibility, included in systematic review, and, if applicable, included in the meta-analysis). | Methods |
| Data collection process | 10 | Describe method of data extraction from reports (e.g., piloted forms, independently, in duplicate) and any processes for obtaining and confirming data from investigators. | Methods and Supplementary Methods 3 |
| Data items | 11 | List and define all variables for which data were sought (e.g., PICOS, funding sources) and any assumptions and simplifications made. | Methods and Supplementary Methods 3 |
| Risk of bias in individual studies | 12 | Describe methods used for assessing risk of bias of individual studies (including specification of whether this was done at the study or outcome level), and how this information is to be used in any data synthesis. | Methods, Supplementary Methods 3 and Supplementary Table 5 |
| Summary measures | 13 | State the principal summary measures (e.g., risk ratio, difference in means). | Methods |
| Synthesis of results | 14 | Describe the methods of handling data and combining results of studies, if done, including measures of consistency (e.g., I^2^) for each meta-analysis. | Methods |
| Risk of bias across studies | 15 | Specify any assessment of risk of bias that may affect the cumulative evidence (e.g., publication bias, selective reporting within studies). | Methods, Supplementary Methods 3 and Supplementary Table 5 |
| Additional analyses | 16 | Describe methods of additional analyses (e.g., sensitivity or subgroup analyses, meta-regression), if done, indicating which were pre-specified. | Methods, Supplementary Methods 1 and Supplementary Tables 6, 7 and 8. |
| **RESULTS** | | | |
| Study selection | 17 | Give numbers of studies screened, assessed for eligibility, and included in the review, with reasons for exclusions at each stage, ideally with a flow diagram. | Results, Figure 1 and Supplementary Methods 2 |
| Study characteristics | 18 | For each study, present characteristics for which data were extracted (e.g., study size, PICOS, follow-up period) and provide the citations. | Table 1 |
| Risk of bias within studies | 19 | Present data on risk of bias for each study and, if available, any outcome-level assessment (see Item 12). | Results and Supplementary Table 5 |
| Results of individual studies | 20 | For all outcomes considered (benefits or harms), present, for each study: (a) simple summary data for each intervention group and (b) effect estimates and confidence intervals, ideally with a forest plot. | Table 2 and Figure 2 |
| Synthesis of results | 21 | Present results of each meta-analysis done, including confidence intervals and measures of consistency. | Results, Table 3, Figure 2, Supplementary Figure 1, Supplementary Tables 7 and 8 |
| Risk of bias across studies | 22 | Present results of any assessment of risk of bias across studies (see Item 15). | Results and Supplementary Table 5 |
| Additional analysis | 23 | Give results of additional analyses, if done (e.g., sensitivity or subgroup analyses, meta-regression [see Item 16]). | Results, Table 3, Figure 2, Supplementary Tables 7 and 8 |
| **DISCUSSION** | | | |
| Summary of evidence | 24 | Summarize the main findings including the strength of evidence for each main outcome; consider their relevance to key groups (e.g., health care providers, users, and policy makers). | Discussion |
| Limitations | 25 | Discuss limitations at study and outcome level (e.g., risk of bias), and at review level (e.g., incomplete retrieval of identified research, reporting bias). | Discussion |
| Conclusions | 26 | Provide a general interpretation of the results in the context of other evidence, and implications for future research. | Discussion |
| **FUNDING** | | | |
| Funding | 27 | Describe sources of funding for the systematic review and other support (e.g., supply of data); role of funders for the systematic review. | Acknowledgments |

This table outlines the PRISMA checklist and is modified from Moher et al. (2009) [11].

# Supplementary Table 3: Reporting checklist for meta-analyses of observational studies

| Reporting of background should include | |
| --- | --- |
| Problem definition | See Introduction |
| Hypothesis statement | See Introduction |
| Description of study outcome(s) | Significant spatial convergence between peak coordinates of GM differences between cases and controls |
| Type of exposure or intervention used | PUI |
| Type of study designs used | Cross-sectional, comparative studies in PUI versus a healthy comparison group |
| Study population | PUI vs control, no specific restrictions |
| Reporting of search strategy should include | |
| Qualifications of searchers (eg, librarians and investigators) | One experienced research assistant and one medical doctor |
| Search strategy, including time period included in the synthesis and keywords | See Methods |
| Effort to include all available studies, including contact with authors | See Methods |
| Databases and registries searched | See Methods |
| Search software used, name and version, including special features used (eg, explosion) | N/A |
| Use of hand searching (eg, reference lists of obtained articles) | See Methods |
| List of citations located and those excluded, including justification | See Supplementary Methods 2 for list of studies excluded in the eligibility assessment stage |
| Method of addressing articles published in languages other than English | Excluded |
| Method of handling abstracts and unpublished studies | Only peer-reviewed publications included |
| Description of any contact with authors | See Methods and Supplementary Methods 3 |
| Reporting of methods should include | |
| Description of relevance or appropriateness of studies assembled for assessing the hypothesis to be tested | Cross-sectional, comparative studies were assembled |
| Rationale for the selection and coding of data (eg, sound clinical principles or convenience) | Standard data selection and coding for ALE meta-analysis |
| Documentation of how data were classified and coded (eg, multiple raters, blinding, and interrater reliability) | Peak coordinates were independently extracted by two researchers. Descriptive data were extracted by one researcher and independently checked by a second researcher. |
| Assessment of confounding (eg, comparability of cases and controls in studies where appropriate) | See Discussion |
| Assessment of study quality, including blinding of quality assessors; stratification or regression on possible predictors of study results | Quality assessors were not blinded. See Results, Discussion, Supplementary Methods 3 and Supplementary Table 5. |
| Assessment of heterogeneity | Subgroup analysis results were suggestive of heterogeneity. See Results and Discussion |
| Description of statistical methods (eg, complete description of fixed or random effects models, justification of whether the chosen models account for predictors of study results, dose-response models, or cumulative meta-analysis) in sufficient detail to be replicated | See Methods |
| Provision of appropriate tables and graphics | See Tables 1-3 and Figures 1-2 |
| Reporting of results should include | |
| Graphic summarizing individual study estimates and overall estimate | See Figure 2 and Supplementary Figure 1 |
| Table giving descriptive information for each study included | See Tables 1-2 |
| Results of sensitivity testing (eg, subgroup analysis) | See Results, Table 3 and Supplementary Tables 7-8 |
| Indication of statistical uncertainty of findings | See Table 3 |
| Reporting of discussion should include | |
| Quantitative assessment of bias (eg, publication bias) | Not carried out, as this is not currently standard practice in ALE meta-analysis (although this is an area under development) [12] |
| Justification for exclusion (eg, exclusion of non-English-language citations) | See Discussion  Non-English language papers were excluded since translation of manuscripts into English would have required access to specialist technical imaging expertise in a variety of languages, for which resourcing was not available in this study. |
| Assessment of quality of included studies | See Results, Discussion, Supplementary Methods 3 and Supplementary Table 5 |
| Reporting of conclusions should include | |
| Consideration of alternative explanations for observed results | See Discussion |
| Generalization of the conclusions (ie, appropriate for the data presented and within the domain of the literature review) | See Discussion |
| Guidelines for future research | See Discussion |
| Disclosure of funding source | See Acknowledgments |

The table outlines the meta-analysis of observational studies in epidemiology (MOOSE) guidance and is modified from Stroup et al. (2000) [13].

ALE = anatomical likelihood estimation; GM = gray matter; PUI = Problematic Usage of the Internet

# Supplementary Table 4: Extracted peak coordinates for each study

| **Study** | **Technique** | **Experiment** | **Subjects, n**^1^ | **Peak coordinates** | | | **Standard space** |
| --- | --- | --- | --- | --- | --- | --- | --- |
|  |  |  |  | **X** | **Y** | **Z** |  |
| Choi et al. (2017) [14] | VBM | IGD<C | 22 | -38 | 24 | 31 | MNI |
| Han et al. (2012) [15] | VBM | OGA>C | 18 | -20 | -29 | 2 | TAL |
|  |  |  |  | -37 | -60 | 35 | TAL |
|  |  | OGA<C | 18 | 59 | -38 | -19 | TAL |
|  |  |  |  | 48 | -71 | -5 | TAL |
|  |  |  |  | -44 | -76 | -4 | TAL |
|  |  |  |  | -47 | -55 | -10 | TAL |
|  |  |  |  | -57 | -27 | -21 | TAL |
| Horvath et al. (2020) [16] | VBM | SPA<C | 22 | 27 | 17 | -5 | MNI |
|  |  |  |  | -27 | -9 | -48 | MNI |
|  |  |  |  | -14 | -36 | -3 | MNI |
|  |  | SPA>C | 22 | -44 | -75 | 9 | MNI |
| Jin et al. (2016) [17] | VBM | IGD<C | 21 | 9 | 26 | 27 | TAL |
|  |  |  |  | -9 | 25 | 23 | TAL |
|  |  |  |  | 8 | 56 | -16 | TAL |
|  |  |  |  | -9 | 58 | -7 | TAL |
|  |  |  |  | 32 | 25 | 38 | TAL |
|  |  |  |  | -34 | 24 | 39 | TAL |
|  |  |  |  | 5 | -3 | 62 | TAL |
| Ko et al. (2015) [18] | VBM | IGD<C | 30 | 35 | -7 | -18 | MNI |
|  |  |  |  | 30 | 5 | -18 | MNI |
|  |  |  |  | -36 | -13 | -14 | MNI |
|  |  |  |  | -24 | -1 | -18 | MNI |
| Lee, Namkoong et al. (2018) [19] | VBM | IGD<C | 30 | 6 | 26 | 21 | MNI |
|  |  |  |  | 2 | 5 | 50 | MNI |
|  |  |  |  | -63 | 3 | 12 | MNI |
|  |  |  |  | -35 | -57 | 42 | MNI |
|  |  |  |  | -32 | 17 | -38 | MNI |
| Lee, Park et al. (2018) [20] | SBM | IGD<C | 35 | 7 | 21 | 53 | TAL |
|  |  |  |  | -10 | 17 | 45 | TAL |
|  |  |  |  | -9 | -30 | 40 | TAL |
|  |  |  |  | -15 | -62 | 61 | TAL |
| Lin et al. (2015) [21] | VBM | IGA<C | 35 | -60 | 50 | -2 | MNI |
|  |  |  |  | 51 | 33 | 16.5 | MNI |
|  |  |  |  | -36 | 23 | 15 | MNI |
|  |  |  |  | 6 | -55 | 55 | MNI |
|  |  |  |  | -9 | -30 | 36 | MNI |
|  |  |  |  | 22.5 | -4.5 | -4.5 | MNI |
|  |  |  |  | -6 | 37.5 | -12 | MNI |
| Seok and Sohn (2018) [22] | VBM | IGD>C | 20 | -8 | 14 | 10 | MNI |
|  |  | IGD<C | 20 | 44 | 51 | 8 | MNI |
|  |  |  |  | -37 | 45 | 20 | MNI |
| Sun et al. (2014) [1] | VBM | IGA>C | 18 | 54 | -19 | -27 | MNI |
|  |  |  |  | 27 | -24 | -24 | MNI |
|  |  |  |  | 48 | -45 | -3 | MNI |
|  |  | IGA<C | 18 | -18 | -28 | 76 | MNI |
| C. Wang et al. (2021) [23] | VBM | IGD<C | 26 | -23 | -6 | 68 | MNI |
|  |  |  |  | -14 | -12 | 62 | MNI |
| S. Wang et al. (2018) [24] | SBM | IGD>C | 32 | 38 | -17 | 1 | MNI |
|  |  |  |  | -40 | -10 | 2 | MNI |
|  |  |  |  | 57 | -47 | -22 | MNI |
|  |  | IGD<C | 32 | 46 | -45 | 12 | MNI |
|  |  |  |  | 19 | -70 | 31 | MNI |
|  |  |  |  | -48 | -35 | -4 | MNI |
|  |  |  |  | 49 | -8 | 32 | MNI |
| Y. Wang et al. (2016) [25] | VBM | MPD<C | 34 | 16.5 | 42 | 54 | MNI |
|  |  |  |  | 60 | 25.5 | 9 | MNI |
|  |  |  |  | -10.5 | 37.5 | 28.5 | MNI |
|  |  |  |  | 13.5 | 43.5 | -9 | MNI |
|  |  |  |  | 31.5 | -88.5 | 18 | MNI |
|  |  |  |  | -13.5 | 46.5 | 10.5 | MNI |
|  |  |  |  | -1.5 | -12 | 9 | MNI |
| Z. Wang et al. (2018) [26] | SBM | IGD>C | 38 | 9.2 | -34.5 | 29.1 | TAL |
|  |  | IGD<C^2^ | 38 | -36.7 | -82.4 | 11.6 | TAL |
|  |  |  |  | -46.6 | -20.1 | 57.4 | TAL |
|  |  |  |  | -45.4 | -4.3 | 14.1 | TAL |
|  |  |  |  | -25.1 | 40.6 | -9.9 | TAL |
|  |  |  |  | -6 | -81.6 | 30.6 | TAL |
|  |  |  |  | 59.9 | -35.4 | -14.5 | TAL |
|  |  |  |  | 25.5 | -75.4 | 33.4 | TAL |
|  |  |  |  | 34.1 | -81.3 | 10.9 | TAL |
|  |  |  |  | 6.4 | -81.5 | 32.8 | TAL |
|  |  |  |  | 55.8 | -55 | 8.5 | TAL |
|  |  |  |  | -61.1 | -14.8 | -1.8 | TAL |
|  |  |  |  | -21.6 | -62.8 | 54.9 | TAL |
|  |  |  |  | 58.4 | -37.2 | 37.5 | TAL |
|  |  |  |  | 45.3 | -40.1 | 9 | TAL |
| Weng et al. (2013) [27] | VBM | OGA<C | 17 | 34 | 25 | -21 | MNI |
|  |  |  |  | -31 | -6 | -3 | MNI |
|  |  |  |  | 37 | -7 | -4 | MNI |
|  |  |  |  | 2 | -4 | 59 | MNI |
| Yoon et al. (2017) [28] | VBM | IGD>C | 19 | 16 | -56 | 34 | MNI |
|  |  |  |  | -34 | -9 | -21 | MNI |
|  |  |  |  | 30 | -4 | -26 | MNI |
| Yuan et al. (2011) [29] | VBM | IAD<C | 18 | -32 | 26 | 36 | MNI |
|  |  |  |  | 27 | 29 | 37 | MNI |
|  |  |  |  | -9 | 25 | 26 | MNI |
|  |  |  |  | -9 | 58 | -15 | MNI |
|  |  |  |  | 17 | 61 | -5 | MNI |
|  |  |  |  | -4 | -8 | 63 | MNI |
|  |  |  |  | 6 | -4 | 63 | MNI |
|  |  |  |  | -27 | -61 | -33 | MNI |
|  |  |  |  | 28 | -56 | -48 | MNI |
| Zhou et al. (2011) [30] | VBM | IA<C | 15 | -10 | 29 | 20 | MNI |
|  |  |  |  | -32 | -56 | 6 | MNI |
|  |  |  |  | -25 | -63 | 5 | MNI |
|  |  |  |  | -19 | -68 | 8 | MNI |
|  |  |  |  | -37 | -29 | 22 | MNI |
|  |  |  |  | -3 | -88 | 11 | MNI |

^1^n refers to sample size of either the PUI or control group, whichever was smaller

^2^Contains data from cortical thickness and cortical volume measurements

C = control; IA = Internet addiction; IAD = Internet addiction disorder; IGA = Internet gaming addiction; IGD = Internet gaming disorder; MNI = Montreal Neurological Institute; MPD = mobile phone dependence; OGA = online game addiction; PUI = Problematic Usage of the Internet; SBM = surface-based morphometry; SPA = smartphone addiction; TAL = Talairach; VBM = voxel-based morphometry.

# Supplementary Table 5: Study quality checklist

|  | Screening tool for PUI | Diagnostic interview | Mood/anxiety disorders | Substance misuse | Impulse control/gambling | ADHD/impulsivity | IQ | Level of education | Sample size >10 per group | Magnet strength ≥1.5 T | MRI slice thickness ≤3 mm | Coordinates reported in standard space | Statistical parameters for significance | Total |
| --- | --- | --- | --- | --- | --- | --- | --- | --- | --- | --- | --- | --- | --- | --- |
| Choi et al. (2017) [14] | 1 | 1 | 1 | 1 | 0 | 1 | 1 | 0 | 1 | 1 | 1 | 1 | 1 | 11 |
| Han et al. (2012) [15] | 1 | 0 | 1 | 1 | 0 | 1 | 0 | 1 | 1 | 1 | 1 | 1 | 1 | 10 |
| Horvath et al. (2020) [16] | 1 | 0 | 0 | 0 | 0 | 1 | 0 | 1 | 1 | 1 | 1 | 1 | 1 | 8 |
| Jin et al. (2016) [17] | 1 | 0 | 0 | 1 | 0 | 0 | 0 | 1 | 1 | 1 | 1 | 1 | 1 | 8 |
| Ko et al. (2015) [18] | 1 | 1 | 1 | 1 | 0 | 1 | 0 | 1 | 1 | 1 | 1 | 1 | 1 | 11 |
| Lee, Namkoong et al. (2018) [19] | 1 | 1 | 1 | 1 | 0 | 1 | 1 | 0 | 1 | 1 | 1 | 1 | 1 | 11 |
| Lee, Park et al. (2018) [20] | 1 | 1 | 1 | 1 | 0 | 1 | 1 | 0 | 1 | 1 | 1 | 1 | 1 | 11 |
| Lin et al. (2015) [21] | 1 | 0 | 1 | 1 | 0 | 0 | 0 | 0 | 1 | 1 | 1 | 1 | 1 | 8 |
| Seok and Sohn (2018) [22] | 1 | 1 | 0 | 0 | 0 | 1 | 0 | 1 | 1 | 1 | 0 | 1 | 1 | 8 |
| Sun et al. (2014) [1] | 1 | 1 | 1 | 1 | 0 | 1 | 0 | 1 | 1 | 1 | 1 | 1 | 1 | 11 |
| C. Wang et al. (2021) [23] | 1 | 0 | 0 | 0 | 0 | 0 | 0 | 0 | 1 | 1 | 1 | 1 | 1 | 6 |
| S. Wang et al. (2018) [24] | 1 | 0 | 1 | 1 | 0 | 0 | 0 | 1 | 1 | 1 | 1 | 1 | 1 | 9 |
| Y. Wang et al. (2016) [25] | 1 | 0 | 0 | 0 | 0 | 1 | 0 | 1 | 1 | 1 | 1 | 1 | 1 | 8 |
| Z. Wang et al. (2018) [26] | 1 | 0 | 1 | 1 | 0 | 0 | 0 | 1 | 1 | 1 | 1 | 1 | 1 | 9 |
| Weng et al. (2013) [27] | 1 | 0 | 0 | 0 | 0 | 1 | 0 | 1 | 1 | 1 | 0^1^ | 1 | 1 | 7 |
| Yoon et al. (2017) [28] | 1 | 0 | 1 | 1 | 0 | 1 | 1 | 0 | 1 | 1 | 1 | 1 | 1 | 10 |
| Yuan et al. (2011) [29] | 1 | 0 | 0 | 1 | 0 | 0 | 0 | 1 | 1 | 1 | 1 | 1 | 1 | 8 |
| Zhou et al. (2011) [30] | 1 | 0 | 1^2^ | 1^2^ | 0 | 0 | 0 | 1^2^ | 1 | 1 | 1 | 1 | 1 | 9 |
| Total | 18 | 6 | 11 | 13 | 0 | 11 | 4 | 12 | 18 | 18 | 16 | 18 | 18 |  |

^1^MRI parameters reported did not include slice thickness for T1-weighted images

^2^Includes information from unpublished sources

ADHD = attention-deficit/hyperactivity disorder; PUI = Problematic Usage of the Internet.

# Supplementary Table 6: Experiments included in each meta-analysis

| **Study** | **Experiment** | **VBM meta-analysis** | **IGD meta-analysis** | **Meta-analysis across diagnoses/modalities** |
| --- | --- | --- | --- | --- |
| Choi et al. (2017) [14] | IGD<C |  |  |  |
| Han et al. (2012) [15] | OGA>C |  |  |  |
|  | OGA<C |  |  |  |
| Horvath et al. (2020) [16] | SPA<C |  |  |  |
|  | SPA>C |  |  |  |
| Jin et al. (2016) [17] | IGD<C |  |  |  |
| Ko et al. (2015) [18] | IGD<C |  |  |  |
| Lee, Namkoong et al. (2018) [19] | IGD<C |  |  |  |
| Lee, Park et al. (2018) [20] | IGD<C |  |  |  |
| Lin et al. (2015) [21] | IGA<C |  |  |  |
| Seok and Sohn (2018) [22] | IGD>C |  |  |  |
|  | IGD<C |  |  |  |
| Sun et al. (2014) [1] | IGA>C |  |  |  |
|  | IGA<C |  |  |  |
| C. Wang et al. (2021) [23] | IGD<C |  |  |  |
| S. Wang et al. (2018) [24] | IGD>C |  |  |  |
|  | IGD<C |  |  |  |
| Y. Wang et al. (2016) [25] | MPD<C |  |  |  |
| Z. Wang et al. (2018) [26] | IGD>C |  |  |  |
|  | IGD<C |  |  |  |
| Weng et al. (2013) [27] | OGA<C |  |  |  |
| Yoon et al. (2017) [28] | IGD>C |  |  |  |
| Yuan et al. (2011) [29] | IAD<C |  |  |  |
| Zhou et al. (2011) [30] | IA<C |  |  |  |
| Total experiments per analysis | | 19 | 18 | 23 |

Gray shading indicates inclusion in the relevant analysis.

C = control; IA = Internet addiction; IAD = Internet addiction disorder; IGA = Internet gaming addiction; IGD = Internet gaming disorder; MPD = mobile phone dependence; OGA = online game addiction; SPA = smartphone addiction; VBM = voxel-based morphometry.

# Supplementary Table 7: Jackknife sensitivity analysis of the three clusters identified in the voxel-based morphometry meta-analysis

| **Study** | **Experiment** | **Chosen min cluster size (mm^3^)** | **Cluster 1** | **Cluster 2** | **Cluster 3** |
| --- | --- | --- | --- | --- | --- |
| Choi et al. (2017) [14] | IGD<C | 648 |  |  |  |
| Han et al. (2012) [15] | OGA>C | 664 |  |  |  |
|  | OGA<C | 640 |  |  |  |
| Horvath et al. (2020) [16] | SPA<C | 616 |  |  |  |
|  | SPA>C | 640 |  |  |  |
| Jin et al. (2016) [17] | IGD<C | 608 |  |  |  |
| Ko et al. (2015) [18] | IGD<C | 656 |  |  |  |
| Lee, Namkoong et al. (2018) [19] | IGD<C | 584 |  |  |  |
| Lin et al. (2015) [21] | IGA<C | 600 |  |  |  |
| Seok and Sohn (2018) [22] | IGD>C | 656 |  |  |  |
|  | IGD<C | 648 |  |  |  |
| Sun et al. (2014) [1] | IGA>C | 640 |  |  |  |
|  | IGA<C | 648 |  |  |  |
| C. Wang et al. (2021) [23] | IGD<C | 640 |  |  |  |
| Y. Wang et al. (2016) [25] | MPD<C | 608 |  |  |  |
| Weng et al. (2013) [27] | OGA<C | 608 |  |  |  |
| Yoon et al. (2017) [28] | IGD>C | 648 |  |  |  |
| Yuan et al. (2011) [29] | IAD<C | 624 |  |  |  |
| Zhou et al. (2011) [30] | IA<C | 640 |  |  |  |
| Total number of iterations in which cluster was found | | | 16 | 16 | 15 |

The voxel-based morphometry meta-analysis was repeated 19 times, leaving out one experiment each time. Gray shading denotes that a cluster was preserved when the corresponding experiment was removed from the analysis.

C = control; IA = Internet addiction; IAD = Internet addiction disorder; IGA = Internet gaming addiction; IGD = Internet gaming disorder; min = minimum; MPD = mobile phone dependence; OGA = online game addiction; SPA = smartphone addiction.

# Supplementary Table 8: Changes in cluster characteristics during jackknife sensitivity analysis

| Cluster | Volume (mm^3^) | Peak MNI coordinates | | | ALE score | *P* value | *Z* score | Hemisphere | Gyrus | BA |
| --- | --- | --- | --- | --- | --- | --- | --- | --- | --- | --- |
|  |  | X | Y | Z |  |  |  |  |  |  |
| 1 | 768-832 | 6 / 4^1^ | -2 / -4^1^ | 62 | .014 | 6.87-9.39 x10^-6^ | 4.28-4.35 | 77-79% R, 21-23% L | 98% medial frontal gyrus,  2% superior frontal gyrus | 100% BA6 |
|  |  | -2 | -6 | 62 | .009 | 1.61-2.02 x10^-4^ | 3.54-3.60 |  |  |  |
| 2 | 760-800 | -10 | 28 | 20 | .017 | 2.57-3.78 x10^-7^ | 4.95-5.02 | 100% L | 68-71% anterior cingulate,  29-32% cingulate gyrus | 67-73% BA32, 27-33% BA24 |
| 3 | 656-704 | -34 | 26 | 36 | .015 | 1.92-2.80 x10^-6^ | 4.53-4.62 | 100% L | 58-60% middle frontal gyrus,  40-42% precentral gyrus | 64-67% BA9, 33-36% BA8 |

Range of values for cluster parameters observed during jackknife analysis for all iterations where a cluster was significant.

^1^Alternative peak coordinate in cluster 1 was observed when Lee, Namkoong et al. (2018) [19], IGD<C was removed from the analysis.

ALE = anatomical likelihood estimation; BA = Brodmann Area; L = left; MNI = Montreal Neurological Institute; R = right.

# References

1. Sun Y, Sun J, Zhou Y, Ding W, Chen X, Zhuang Z, et al. Assessment of in vivo microstructure alterations in gray matter using DKI in internet gaming addiction. Behav Brain Funct. 2014;10:37.

2. Ioannidis K, Hook R, Goudriaan AE, Vlies S, Fineberg NA, Grant JE, et al. Cognitive deficits in problematic internet use: meta-analysis of 40 studies. Br J Psychiatry. 2019;215:639–646.

3. Evans AC, Collins DL, Mills SR, Brown ED, Kelly RL, Peters TM. 3D statistical neuroanatomical models from 305 MRI volumes. 1993 IEEE Conf. Rec. Nucl. Sci. Symp. Med. Imaging Conf., San Francisco, CA, USA: IEEE; 1993. p. 1813–1817.

4. Talairach J, Tournoux P. Co-planar stereotaxic atlas of the human brain: 3-dimensional proportional system: an approach to cerebral imaging. Stuttgart: Georg Thieme Verlag; 1988.

5. Turkeltaub PE, Eickhoff SB, Laird AR, Fox M, Wiener M, Fox P. Minimizing within-experiment and within-group effects in activation likelihood estimation meta-analyses. Hum Brain Mapp. 2012;33:1–13.

6. Eickhoff SB, Laird AR, Grefkes C, Wang LE, Zilles K, Fox PT. Coordinate-based activation likelihood estimation meta-analysis of neuroimaging data: A random-effects approach based on empirical estimates of spatial uncertainty. Hum Brain Mapp. 2009;30:2907–2926.

7. Eickhoff SB, Bzdok D, Laird AR, Kurth F, Fox PT. Activation likelihood estimation meta-analysis revisited. Neuroimage. 2012;59:2349–2361.

8. Du M, Liu J, Chen Z, Huang X, Li J, Kuang W, et al. Brain grey matter volume alterations in late-life depression. J Psychiatry Neurosci. 2014;39:397–406.

9. Shepherd AM, Matheson SL, Laurens KR, Carr VJ, Green MJ. Systematic meta-analysis of insula volume in schizophrenia. Biol Psychiatry. 2012;72:775–784.

10. Müller VI, Cieslik EC, Laird AR, Fox PT, Radua J, Mataix-Cols D, et al. Ten simple rules for neuroimaging meta-analysis. Neurosci Biobehav Rev. 2018;84:151–161.

11. Moher D, Liberati A, Tetzlaff J, Altman DG. Preferred Reporting Items for Systematic Reviews and Meta-Analyses: The PRISMA Statement. PLoS Med. 2009;6:e1000097.

12. Acar F, Seurinck R, Eickhoff SB, Moerkerke B. Assessing robustness against potential publication bias in Activation Likelihood Estimation (ALE) meta-analyses for fMRI. PLoS One. 2018;13:e0208177.

13. Stroup DF, Berlin JA, Morton SC, Olkin I, Williamson GD, Rennie D, et al. Meta-analysis of observational studies in epidemiology: a proposal for reporting. JAMA. 2000;283:2008–2012.

14. Choi J, Cho H, Kim JY, Jung DJ, Ahn KJ, Kang HB, et al. Structural alterations in the prefrontal cortex mediate the relationship between Internet gaming disorder and depressed mood. Sci Rep. 2017;7:1245.

15. Han DH, Lyoo IK, Renshaw PF. Differential regional gray matter volumes in patients with on-line game addiction and professional gamers. J Psychiatr Res. 2012;46:507–515.

16. Horvath J, Mundinger C, Schmitgen MM, Wolf ND, Sambataro F, Hirjak D, et al. Structural and functional correlates of smartphone addiction. Addict Behav. 2020;105:106334.

17. Jin C, Zhang T, Cai C, Bi Y, Li Y, Yu D, et al. Abnormal prefrontal cortex resting state functional connectivity and severity of internet gaming disorder. Brain Imaging Behav. 2016;10:719–729.

18. Ko CH, Hsieh TJ, Wang PW, Lin WC, Yen CF, Chen CS, et al. Altered gray matter density and disrupted functional connectivity of the amygdala in adults with Internet gaming disorder. Prog Neuropsychopharmacol Biol Psychiatry. 2015;57:185–192.

19. Lee D, Namkoong K, Lee J, Jung YC. Abnormal gray matter volume and impulsivity in young adults with Internet gaming disorder. Addict Biol. 2018;23:1160–1167.

20. Lee D, Park J, Namkoong K, Kim IY, Jung YC. Gray matter differences in the anterior cingulate and orbitofrontal cortex of young adults with Internet gaming disorder: Surface-based morphometry. J Behav Addict. 2018;7:21–30.

21. Lin X, Dong G, Wang Q, Du X. Abnormal gray matter and white matter volume in ‘Internet gaming addicts’. Addict Behav. 2015;40:137–143.

22. Seok JW, Sohn JH. Altered gray matter volume and resting-state connectivity in individuals with internet gaming disorder: A voxel-based morphometry and resting-state functional magnetic resonance imaging study. Front Psychiatry. 2018;9:77.

23. Wang C, Zhang Z, Che L, Wu Y, Qian H, Guo X. The gray matter volume in superior frontal gyrus mediates the impact of reflection on emotion in Internet gaming addicts. Psychiatry Res Neuroimaging. 2021;310:111269.

24. Wang S, Liu J, Tian L, Chen L, Wang J, Tang Q, et al. Increased insular cortical thickness associated with symptom severity in male youths with internet gaming disorder: A surface-based morphometric study. Front Psychiatry. 2018;9:99.

25. Wang Y, Zou Z, Song H, Xu X, Wang H, D’Oleire Uquillas F, et al. Altered gray matter volume and white matter integrity in college students with mobile phone dependence. Front Psychol. 2016;7:597.

26. Wang Z, Wu L, Yuan K, Hu Y, Zheng H, Du X, et al. Cortical thickness and volume abnormalities in Internet gaming disorder: Evidence from comparison of recreational Internet game users. Eur J Neurosci. 2018;48:1654–1666.

27. Weng CB, Qian RB, Fu XM, Lin B, Han XP, Niu CS, et al. Gray matter and white matter abnormalities in online game addiction. Eur J Radiol. 2013;82:1308–1312.

28. Yoon EJ, Choi JS, Kim H, Sohn BK, Jung HY, Lee JY, et al. Altered hippocampal volume and functional connectivity in males with Internet gaming disorder comparing to those with alcohol use disorder. Sci Rep. 2017;7:5744.

29. Yuan K, Qin W, Wang G, Zeng F, Zhao L, Yang X, et al. Microstructure abnormalities in adolescents with internet addiction disorder. PLoS One. 2011;6:e20708.

30. Zhou Y, Lin FC, Du YS, Qin L Di, Zhao ZM, Xu JR, et al. Gray matter abnormalities in internet addiction: A voxel-based morphometry study. Eur J Radiol. 2011;79:92–95.
